# Field performance of transgenic citrus trees: Assessment of the long-term expression of *uidA* and *nptII* transgenes and its impact on relevant agronomic and phenotypic characteristics

**DOI:** 10.1186/1472-6750-12-41

**Published:** 2012-07-15

**Authors:** Elsa Pons, Josep E Peris, Leandro Peña

**Affiliations:** 1 Centro de Protección Vegetal y Biotecnología, Instituto Valenciano de Investigaciones Agrarias (IVIA), 46113 Moncada, Valencia, Spain

**Keywords:** Citrus, Transgenic trees, Selectable marker genes, *uidA*, *nptII*, Substantial equivalence, Pleiotropic effects, Long-term transgene stability, Phenotypic assessment, Field performance.

## Abstract

**Background:**

The future of genetic transformation as a tool for the improvement of fruit trees depends on the development of proper systems for the assessment of unintended effects in field-grown GM lines. In this study, we used eight transgenic lines of two different citrus types (sweet orange and citrange) transformed with the marker genes β-glucuronidase (*uidA*) and neomycin phosphotransferase II (*nptII*) as model systems to study for the first time in citrus the long-term stability of transgene expression and whether transgene-derived pleiotropic effects occur with regard to the morphology, development and fruit quality of orchard-grown GM citrus trees.

**Results:**

The stability of the integration and expression of the transgenes was confirmed in 7-year-old, orchard-grown transgenic lines by Southern blot analysis and enzymatic assays (GUS and ELISA NPTII), respectively. Little seasonal variation was detected in the expression levels between plants of the same transgenic line in different organs and over the 3 years of analysis, confirming the absence of rearrangements and/or silencing of the transgenes after transferring the plants to field conditions. Comparisons between the GM citrus lines with their non-GM counterparts across the study years showed that the expression of these transgenes did not cause alterations of the main phenotypic and agronomic plant and fruit characteristics. However, when comparisons were performed between diploid and tetraploid transgenic citrange trees and/or between juvenile and mature transgenic sweet orange trees, significant and consistent differences were detected, indicating that factors other than their transgenic nature induced a much higher phenotypic variability.

**Conclusions:**

Our results indicate that transgene expression in GM citrus remains stable during long-term agricultural cultivation, without causing unexpected effects on crop characteristics. This study also shows that the transgenic citrus trees expressing the selectable marker genes that are most commonly used in citrus transformation were substantially equivalent to the non-transformed controls with regard to their overall agronomic performance, as based on the use of robust and powerful assessment techniques. Therefore, future studies of the possible pleiotropic effects induced by the integration and expression of transgenes in field-grown GM citrus may focus on the newly inserted trait(s) of biotechnological interest.

## Background

Crop improvement *via* genetic modification (GM) remains controversial, with one of the major issues being the potential for unintended effects caused by the integration and expression of the transgene. Such unintended effects may occur as a result of interactions between the transgene or its regulatory elements and the plant genome at the site of insertion. The integration site could affect a transgenic plant in two ways: with regard to the functioning of the surrounding DNA sequences (insertion effect) and with regard to the expression of the transgene (position effect). The insertion effect can be of a mutagenic nature and could result in null, loss of function, gain of function or other possible phenotypes, depending on the specific DNA region that is randomly targeted by the insertion and the regulatory elements within the foreign DNA (T-DNA in the case of *Agrobacterium tumefaciens*-mediated transformation). With respect to the position effect, it is well known that the integration site and transgene architecture (i.e., transgene copy number) may influence the transgene expression level and stability (see [1,2] for reviews). All of these effects can vary according to the specific integration event and would, therefore, be unique to each independent transgenic line. Moreover, the full range of recurring locus-independent changes induced by the expression of a given transgene constitutes the so-called pleiotropic effects. Although some of these effects may be expected based on the intended trait, others may occur through unexpected interactions of the transgene products with plant cell metabolism [3].

Within the context of GM crops, the relevance of unintended effects is mainly related to their implications regarding agronomic performance [4]. There are examples showing that transgenesis may generate non-desirable phenotypic alterations as a consequence of pleiotropic changes in plant growth and development, compromising the preservation of the identity of the transformed genotype [5-9]. Although the existence of such unintended effects does not necessarily generate concerns in terms of safety (for human health and/or the environment), it is important to evaluate their extent to validate or discard the application of each genetic engineering product in agriculture [10]. Some studies have reported unintended pleiotropic effects generated by the expression of selectable marker genes [11-13], despite the fact that such transgenes are not generally believed to alter biological processes in plants [14-16]. These findings indicate that the pleiotropic effects associated with selectable marker genes also need to be assessed in a range of plants, particularly in those that are expected to remain in the field for many years and be subjected to highly variable environmental conditions.

The significance of unintended changes is negligible in most cases because most event-specific effects are routinely eliminated during the early screening stages [1]. However, even after selection, there are some reports of apparently normal transgenic plants exhibiting aberrant behavioral or biochemical characteristics upon further analysis (for reviews, see the references in [17-19]). Such studies often focus on the possibility that a transgene may not result in the desired phenotypic effect when GM plants are moved from a controlled glasshouse environment to more variable field conditions [20]. However, some studies have also reported potentially unintended phenotypic effects of transgenes in GM plants exposed to a range of realistic environmental conditions. Examples of these unexpected traits include lower yields [21-23], an enhanced susceptibility to pathogens [24], altered insect resistance as a consequence of non-targeted changes in secondary metabolism [25] and an enhanced outcrossing ability of transgenic plants [26,27].

Therefore, it is important to investigate the substantial equivalence of transgenic crops through the assessment of phenotypic differences between GM lines and their non-GM counterparts in field trials [4]. The comparative analysis of physiological characteristics, such as agronomic-, morphology- and development-related traits, is an essential first step in identifying these differences [28]. Furthermore, an appropriate experimental design is required for the assessment of GM crops [29]. The guidelines described internationally for performing agronomic and phenotypic analyses of GM crops emphasize that choosing appropriate comparators and performing adequate field trials (e.g., number of growing seasons, replicates, selection of characteristics to be analyzed) are crucial to ensure confidence in the results. The experimental design should also be consistent with the intended method of statistical analysis [30].

Citrus is the most economically important and extensively grown fruit tree crop in the world [31]. Genetic transformation is considered an essential tool in some current citrus improvement programs and offers great opportunities to achieve the goals of interest, such as the resistance to devastating diseases or enhancement of health-promoting fruit qualities [32]. However, there are no available reports regarding the agronomic performance of transgenic citrus plants. Although there are some studies on the integration patterns, expression and inheritance of transgenes in citrus plants and their progeny [33,34], none of these investigations has addressed the impact of transgene integration and expression on agronomic characteristics. The aim of the present study was to estimate the effects of transgenesis on the performance of citrus trees grown in an orchard since 1997 and to study the stability of transgene integration and expression. The experiment involved the release of 8 independent transgenic lines of Carrizo citrange (*Citrus sinensis* L. Osb. X *Poncirus trifoliata* L. Raf.) and Pineapple sweet orange (*C. sinensis* L. Osb.) carrying the marker transgenes *nptII* and *uidA* (GUS). We also included non-transgenic regenerants obtained from the transformation experiments, which were used as the non-GM controls. Making use of comparative analyses of fruit quality, tree morphology and phenology conducted over several years, the present work evaluates the substantial equivalence of field-grown transgenic citrus plants relative to their non-GM counterparts. Furthermore, to validate the evaluation techniques applied in this work, the effects of genetic and physiological factors (other than ‘transgene’) were also investigated. For this purpose, some transgenic lines of each citrus type that could be distinguished by an additional trait, either their ploidy level (diploid vs. tetraploid, in the case of Carrizo citrange) or developmental stage (juvenile vs. mature, in the case of Pineapple orange), served as comparators to test the effects of ‘ploidy’ and ‘ontogeny’ on the parameters studied for the citrange and sweet orange lines, respectively. This is the first detailed study demonstrating the substantial equivalence of field-grown GM and non-GM citrus trees reported thus far and could represent a model for investigating the performance of GM fruit trees under field conditions through the use of appropriate controls and comparators.

## Results

**Figure 1:**
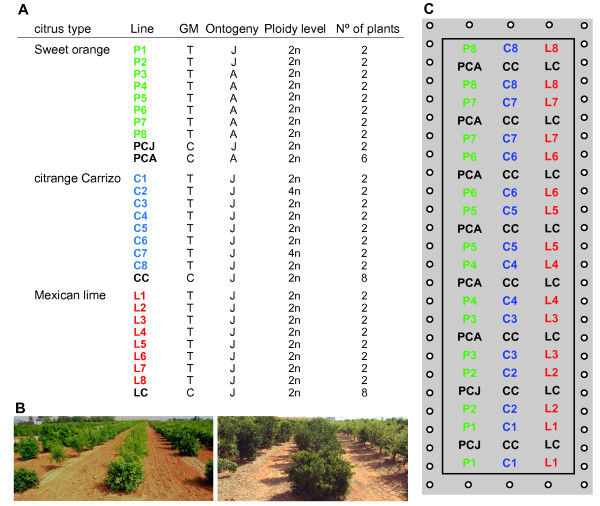
**Experimental field trial (T plot). A**) Description of all of the citrus lines selected for release in the experimental orchard, including the citrus type, genetic modification (GM), developmental stage (ontogeny), ploidy level and number of plants of each line. T, transgenic; C, control; J, juvenile; A, adult; 2n, diploid; 4n, tetraploid. **B**) Images showing the T plot in 1998 (left) and in 2004 (right). **C**) Schematic diagram of the T plot showing the arrangement of the 130 trees, including 16 transgenic plants of Pineapple sweet orange (green), 16 transgenic plants of Carrizo citrange (blue) and 16 transgenic plants of Mexican lime (red). In addition, there were 8 non-transgenic control plants from each citrus type interspersed individually between the two plants from each transgenic line (black). Fifty-eight non-transgenic Clemenules clementine trees planted along an external edge (white circles) were used as a buffer to prevent transgene flow through pollen dispersal.

All of the citrus plants used in the field trial (T plot) were generated previously in our laboratory, and their main characteristics are summarized in Figure 1. The Pineapple sweet orange plants were obtained from the experiments described in Cervera *et al*., 1998a [35]. For the field release, we selected six independent transgenic lines (designated P3 to P8) and one non-GM regenerant (PCA) derived from adult plant material. Moreover, to address the ‘ontogeny’ effect in the sweet orange lines, we also included two independent transgenic lines (designated P1 and P2) and a non-GM control (designated PCJ) derived from juvenile material in the experimental orchard. “Juvenile” transformants flowered in 2002 and set fruits in 2003 for the first time. Although they could not be considered strictly juvenile from that moment on, these plants passed through a transition phase [36] characterized for tree vigorous growth, thorniness, alternate bearing and reduced yield, which prolonged at least for the 3 years of study. Conversely, mature transformants set fruits soon after being grafted in the field and they showed typical features of true-to-type Pineapple sweet orange trees bearing regular fruits. The Carrizo citrange plants were generated in experiments described in Peña *et al*., 1995 [37] and Cervera *et al*., 1998b [38], and six independent transformants were selected for the field release (designated C1, C3, C4, C5, C6 and C8) in addition to one non-GM regenerant. All of these lines were diploid and presented a normal appearance in a preliminary screen under greenhouse conditions [33]. We also decided to include two unintentionally obtained off-type transgenic tetraploid lines (designated C2 and C7) in the field trial to assess the ‘ploidy’ effect in the citrange lines. We did not include a non-GM tetraploid control line because none was spontaneously generated during the course of the original experiments [33]. Mexican lime (*C. aurantifolia* (Christm.) Swing.) plants present in the orchard were obtained from experiments described in [39]. Although our original intention was to conduct the same analyses with these transgenic lime trees, they were excluded from further analysis because they suffered severe symptoms from frost during successive winters.

In 2004, seven years after planting in the orchard (Figure 1B), when all of the transgenic and control lines had experienced several cycles of fruit production, the molecular and phenotypic analyses of each plant were initiated.

### Long-term stability of transgene integration and expression

To demonstrate the long-term stable integration and expression of *nptII* and *uidA* gene cassettes, analyses of genomic DNA were performed on 7-year-old, orchard-grown transgenic citrus trees, and the results were compared to the results previously reported by our group [33,35,38]. Southern blot analysis confirmed the presence of stably integrated transgene cassettes into the plant genomes of all of the transgenic trees. Digestion with either Hin*d*III or *Dra*I + *Cla*I resulted in the generation of internal fragments of the *uidA* and *nptII* cassettes, with the expected sizes of 2.8 and 2.0 kb, respectively. The corresponding non-transgenic controls showed no hybridization signals (results not shown). The T-DNA of the binary vector used has unique restriction sites for *Eco*RI and *Dra*I at the left and right borders of the sequence, respectively, and digestion of the DNA with either of these enzymes generated unique fragments between the T-DNA and plant DNA. A different number of insertions and integration patterns were revealed in the different transgenic lines following hybridization with the *uidA* or *nptII* probes, as summarized in Table 1. All of the transgenic plants exhibited the long-term stable integration of both the *uidA* and *nptII* genes, with different hybridization patterns being detected among independent transgenic lines. As shown in Table 1, the estimated number of copies of each transgene was identical to that shown previously by our group (when the transformants were generated).

**Table 1:** Long-term stability of the integration of transgenes in transgenic sweet orange and citrange lines determined by Southern blot analysis

**Line**	**Copy number determined by Southern blot**			
	**In previous analyses**^**1**^		**In 2004**^**2**^	
	***uidA***	***nptII***	***uidA***	***nptII***
P1	nd	nd	4	4
P2	nd	nd	1	1
P3	2	1	2	1
P4	1	2	1	2
P5	1	3	1	3
P6	1	1	1	1
P7	4	4	4	4
P8	1	1	1	1
C1	2	2	2	2
C2	1	1	1	1
C3	nd	nd	2	2
C4	2	2	2	2
C5	2	1	2	1
C6	2	1	2	1
C7	1	1	1	1
C8	1	1	1	1

^1^Analyses performed prior to the release in the experimental orchard in 1997, as described in [35][38] and [33].

^2^Analyses performed in this work.

nd, not determined.

**Figure 2:**
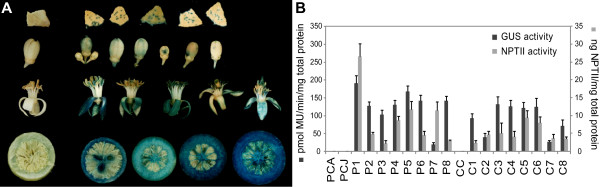
**Characterization of 7-year-old, orchard-grown transgenic citrus trees: analyses of GUS and NPTII protein activities. A**) Histochemical GUS analysis of different organs (*row 1*, leaves; *row 2*, flowers in pre-anthesis; *row 3*, flowers in post-anthesis; *row 4*, transverse sections of immature fruit) from the transgenic citrus plants. Representative image showing the staining patterns exhibited by the different transgenic lines under investigation. *Left column*, control samples showing no coloration; all of the leaves were punched to facilitate substrate infiltration. After the reaction, the organs were cleared of chlorophyll by means of an ethanol series. **B**) GUS and NPTII activities in the leaf samples from all of the sweet orange and citrange lines grown in the experimental orchard. Data represent the average values ± SEM from the different plants of each line, assayed at four time points (seasonally) over the course of one year.

GUS analyses of different organs of the transgenic trees were performed periodically beginning in 2004. All of the transgenic samples showed blue staining in histochemical assays during the 3 consecutive years of the study (Figure 2A), whereas no coloration was visible in the control samples (Figure 2A, left column of the image). In spite of some detectable variation in the expression levels among the different transgenic lines, GUS expression remained relatively high in all of the tissues analyzed for all of the transgenic lines. Moreover, the transgenic plants showed similar conserved patterns of GUS expression throughout the study period, and no drastic decreases or increases in transgene activity were observed within any tree or between trees of the same line.

To estimate the enzyme activities, fluorimetric GUS assays and NPTII ELISAs were performed on leaf samples from all of the plants every 3 months over a period of one year (2007). The measurements were performed using different plants of the same line and at different time points to ensure a reliable representation of the temporal intraline transgene expression. The results regarding the fluorimetric GUS activity and immunological quantification of NPTII accumulation are shown in Figure 2B. All of the transgenic lines displayed both NPTII and GUS activity, with the expression levels varying from 2.8 to 26.6 ng NPTII per mg total protein and from 20.4 to 191.8 pmol MU per min per μg total protein, respectively, in the transgenic samples. These ranges in activity were similar to those obtained in the initial populations of transformants from which we propagated the sweet orange and citrange plants under investigation [33,35]. The data shown in Figure 2B are the average annual values per line. The relatively low SE bars indicate little variation in the expression levels between the plants of the same line and a lack of considerable seasonal fluctuations.

### Morphological and phenological analyses revealed the normal appearance and development of 7-year-old orchard-grown transgenic trees

We performed morphological and phenological analyses of the transgenic trees in comparison with their respective non-GM counterparts to study the influence of transgenesis on the main phenotypic characteristics of the plants (i.e., the ‘transgene’ effect). For this purpose, each citrus genotype was analyzed separately.

**Figure 3:**
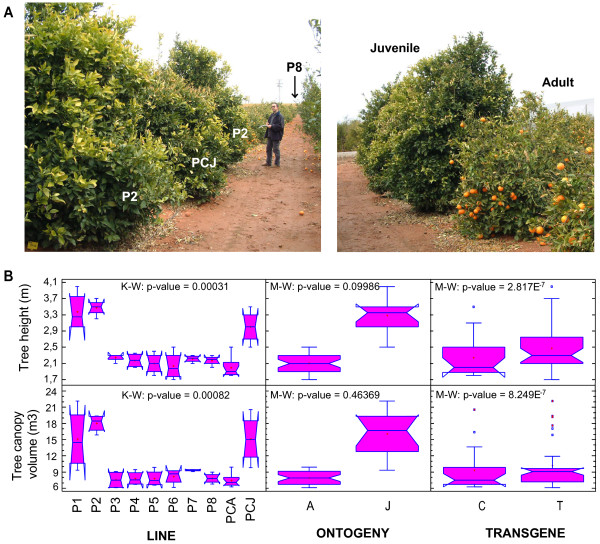
**Morphological analysis of sweet orange trees. A)** Images showing the appearance of the sweet orange trees in the experimental orchard. *Image on the left:* general view of the sweet orange trees (from the P2 to P8 lines) distributed in a row in the orchard; *image on the right:* size comparison between juvenile and adult trees. **B)** Effects of the ‘line’, ‘transgene’ and ‘ontogeny’ factors on the morphological variables Tree height and Tree canopy volume. The data represented in the notched box-and-whisker plots were calculated from measurements recorded over the course of two years (2004 and 2005) at the end of the growing season. K-W, Kruskal-Wallis test (*n* = 48); M-W, Mann–Whitney tests; A, all adult lines (*n* = 36); J, all juvenile lines (*n* = 12); C, all control lines (*n* = 16); T, all transgenic lines (*n* = 32).

Based on an initial visual scrutiny, noticeable differences were detected among the sweet orange lines with respect to the size of the tree, as the juvenile lines showed a greater size than the adult lines (Figure 3A). No other morphological differences were observed among the lines. Indeed, the transgenic trees could not be visually distinguished from their respective non-transgenic controls at any time during the growing season or after fruit harvesting (Figure 1B). To confirm these observations, two morphological variables related to tree size, tree height (TH) and tree canopy volume (TCV), were measured in two consecutive years (2004 and 2005) for each line, and the data were analyzed statistically. Differences among the sweet orange lines were confirmed using the Kruskal-Wallis test (p < 0.001) for the variables TH and TCV. Notched box-and-whisker plots showed that the median values of both variables were always higher for the juvenile lines (PCJ, P1 and P2) than for the adult lines (PCA, P3, P4, P5, P6, P7 and P8), indicating that the factor ‘ontogeny’ (developmental stage) had a marked effect on the parameters. Mann–Whitney tests confirmed the highly significant differences in these variables between ‘ontogeny’ classes (juvenile versus adult lines). However, no significant differences (p < 0.05) in these variables were detected between the transgenic and control lines (Figure 3B). These results indicated that the juvenile plants continued to display the morphological features typical of juvenility (faster growth behavior than adults), even after entering the fruit production stage. In contrast, transgenesis did not affect any of the morphological traits.

**Figure 4:**
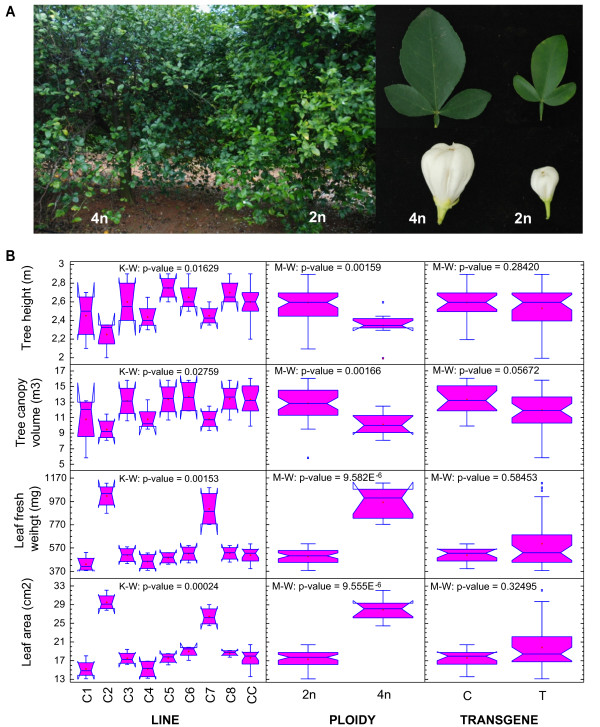
**Morphological analysis of citrange trees. A**) Images showing morphological differences observed between tetraploid (4n) and diploid (2n) lines. *Image on the left:* differences in the coloration and leaf density of the trees; *image on the right:* differences in the morphology of their leaves and flowers. **B**) The effects of the ‘line’, ‘transgene’ and ‘ploidy’ factors on the morphological variables Tree height, Tree canopy volume, Leaf fresh weight and Leaf area. The data represented in the notched box-and-whisker plots were calculated from measurements recorded over the course of two years (2004 and 2005) at the end of the growing season. K-W, Kruskal-Wallis test (*n* = 48); M-W, Mann–Whitney tests; 2n, all diploid lines (*n* = 40); 4n, all tetraploid lines (*n* = 8); C, control line CC (*n* = 16); T, all transgenic lines, except tetraploids C2 and C7 (*n* = 24).

In the citrange population, several obvious differences were visually detected only in lines C2 and C7. These tetraploid lines developed thicker and broader leaves, having a darker green color, and larger flowers and showed a slightly smaller tree size and leaf density in comparison with the rest of the citrange lines (all diploid) (Figure 4A). A Kruskal-Wallis test confirmed significant differences (at the 95% confidence level) among the medians of the variables TH, TCV, leaf fresh weight (LFW) and leaf area (LA) for the citrange lines. Subsequently, Mann–Whitney tests detected a statistically significant ‘ploidy’ effect for the TH and TCV variables (p < 0.01) and for the LFW and LA variables at higher levels of significance (p < 0.0001), whereas the ‘transgene’ factor had no effect on any of these variables (p < 0.05).

**Figure 5:**
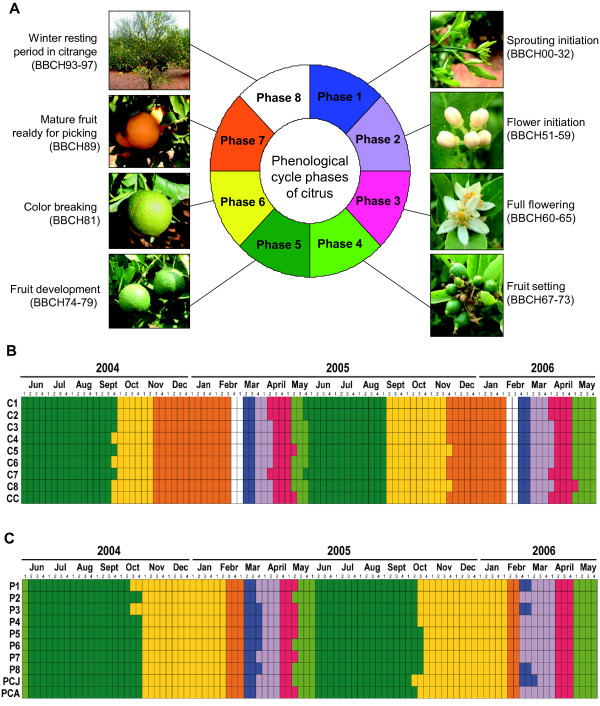
**Phenological assessment. A**) Schematic representation of the phenological cycle of the citrus lines. The main phases of development are shown using different colors (key legend), which were used to draw the phenological calender of **B**) the Carrizo citrange lines and **C**) the Pineapple sweet orange lines. Phenological stages were recorded weekly according to the BBCH codification for citrus and grouped into 8 phases stressing flower and fruit developmental stages.

Phenological calenders showed no differences in the transgenic trees when compared with the non-GM controls for either of the two genotypes studied. Marked differences were not detected due to either ‘ploidy’ (in the citrange lines) or ‘ontogeny’ (in the sweet orange lines). As expected, the most notable differences were observed when the phenological cycles of the two citrus genotypes under study were compared (Figure 5). Therefore, transgenesis *per se* did not affect the morphological appearance or phenological cycle of the trees, whereas other factors, such as the developmental stage (for the sweet orange plants) or the ploidy level (for the citrange plants), had a highly significant impact on the morphological variables.

### The ‘transgene’ effect did not influence fruit quality, whereas ‘ontogeny’ and ‘ploidy’ did alter many quality parameters

To assess whether transgenesis affected the agronomic performance of the transgenic citrus trees, the typical parameters commonly used to define the quality of citrus fruit [40] were evaluated in the fruit samples from the orchard-grown transgenic citrus lines and from their respective non-GM controls. The parameters evaluated for all of the sweet orange and citrange lines in the 2004, 2005 and 2006 seasons (S1 to S3) were as follows: fruit weight (W), fruit volume (V), caliber, the color index (CI), juice content (JC), total soluble solids (TSS), titratable acidity (TA) and maturity index (MI). The fruit of the citrange lines was analyzed for an additional season (2007; S4). The data for each citrus type and season was analyzed separately using an ANOVA procedure to test the effect of ‘line’ on each fruit quality parameter. The ‘transgene’ effect was assessed by performing *a posteriori* contrasts in which each transgenic line was compared with its respective non-GM control. Moreover, as it is known that the developmental stage and the ploidy level of citrus plants may affect the quality of their fruit, the effects of ‘ontogeny’ and ‘ploidy’ were also evaluated in the sweet orange and citrange lines, respectively, by performing the pertinent planned (or *a priori*) contrasts.

**Figure 6:**
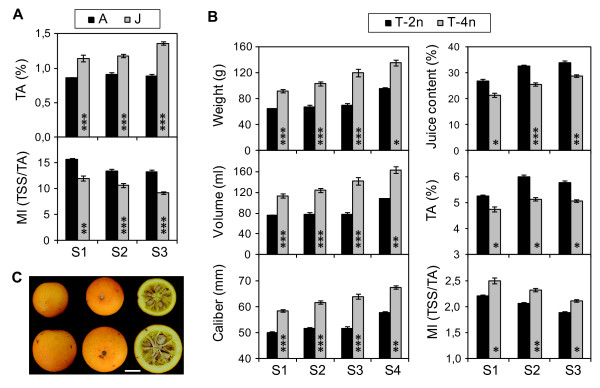
**Graphic representation of the significant and consistent effects detected in the analysis of fruit quality in the sweet orange and citrange lines. A**) ‘ontogeny’ effect detected in sweet orange lines. **B**) ‘Ploidy’ effect detected in citrange lines. **C**) Images showing the representative appearance of mature diploid (2n) and tetraploid (4n) fruit from Carrizo citrange trees. The scale bar represents 2 cm. Level of significance achieved in the planned contrasts: *p < 0.01; **p < 0.001; ***p < 0.0001. Seasons analyzed: S1, season 2004; S2, season 2005; S3, season 2006; S4, season 2007. Average ± SE from contrasts showing significant differences (p < 0.01) in at least two seasons are represented.

A summary of the quality characteristics of the fruit from the sweet orange trees is presented in 1. We observed visually marked variations in yield among the sweet orange trees, depending on the year of analysis. This phenomenon, known as alternancy, is common in such citrus cultivars as Pineapple sweet orange and may affect fruit quality [41]. The lines in which the reduction of the yield was particularly drastic (less than 30 fruits per tree) were PCJ, P1 and P2 for S2 and PCA, P3 and P4 for S3 (shown in bold in Additional file 1). These productivity data were taken into account when drawing conclusions in the analysis of the fruit. The effects of the ‘line’, ‘transgene’ and ‘ontogeny’ factors on the fruit quality of the sweet orange lines are represented in Table 2. The ANOVA results showed that the ‘line’ factor had a significant effect on the variables TA and MI in all of the seasons analyzed. For TA, these effects were always highly significant (p < 0.0001). The ‘line’ factor also had an effect on the parameters caliber, CI and JC but only in one of the three years tested and at a lower significance level (p < 0.01). The ‘line’ factor had no effect on any other variable. The results of the contrasts performed to test the ‘transgene’ effect showed that no significant differences (p < 0.01) were found for any fruit quality parameter evaluated when each transgenic line was compared with its corresponding non-GM control. The only exceptions were the significant differences found for the variable MI detected in the contrasts “P3 vs. PCA” and “P4 vs. PCA” for S3. These results could be explained by the poor yield of the P3 and P4 trees in that particular season (see Additional file 1). Therefore, ‘transgene’ did not induce any detectable difference in fruit quality in the sweet orange lines. Table 2 also shows the results from contrasts performed to test the ‘ontogeny’ effect on the fruit quality parameters in the sweet orange lines. Highly significant differences were detected between the juvenile and adult lines, irrespective of their transgenic nature, for the variables TA and MI in at least two of the three seasons analyzed. As presented in Figure 6A, the juvenile lines showed higher TA (at a p < 0.0001 significance level) and lower MI (at a p < 0.001 significance level) values than the adult lines in all three of the seasons. This result was somewhat expected, taking into account that the differences in the TSS were not detected when comparing juvenile and adult lines (Table 2). In contrast, for W, V, caliber and CI, a significant ‘ontogeny’ effect was only detected for S2 (Table 2), which could be explained by the low yield in all of the juvenile lines in that particular year (see the sampling data in Additional file 1). Thus, ‘transgene’ had no impact on any fruit quality parameter evaluated, whereas a significant and consistent ‘ontogeny’ effect was detected for certain variables. Juvenile transformants were not producing regular fruits five years after flowering for the first time. This should be taken into account when using juvenile instead of mature tissues as starting material for genetic engineering.

**Table 2:** The effects of the ‘line’, ‘transgene’ and ‘ontogeny’ factors on the fruit quality in the sweet orange lines

**Source**	**Fruit quality parameter (dependent variable)**																							
	**Weight**			**Volume**			**Caliber**			**Color Index**			**Juice content**^**1**^			**TA**			**TSS**			**MI**		
	**S1**	**S2**	**S3**	**S1**	**S2**	**S3**	**S1**	**S2**	**S3**	**S1**	**S2**	**S3**	**S1**	**S2**	**S3**	**S1**	**S2**	**S3**	**S1**	**S2**	**S3**	**S1**	**S2**	**S3**
*Factor / ANOVA*^2^																								
Line	NS	NS	NS	NS	NS	NS	NS	*	NS	NS	*	NS	*	NS	NS	***	***	***	NS	NS	NS	*	*	***
Plant (Line)	***	***	***	***	***	***	***	***	***	***	***	***	*	*	NS	***	***	***	***	***	***	***	***	NS
*Contrasts to test ‘transgene’ effect*^3^																								
P3A vs PCA	NS	NS	NS	NS	NS	NS	NS	NS	NS	NS	NS	NS	NS	NS	NS	NS	NS	NS	NS	NS	NS	NS	NS	*
P4A vs PCA	NS	NS	NS	NS	NS	NS	NS	NS	NS	NS	NS	NS	NS	NS	NS	NS	NS	NS	NS	NS	NS	NS	NS	**
P5A vs PCA	NS	NS	NS	NS	NS	NS	NS	NS	NS	NS	NS	NS	NS	NS	NS	NS	NS	NS	NS	NS	NS	NS	NS	NS
P6A vs PCA	NS	NS	NS	NS	NS	NS	NS	NS	NS	NS	NS	NS	NS	NS	NS	NS	NS	NS	NS	NS	NS	NS	NS	NS
P7A vs PCA	NS	NS	NS	NS	NS	NS	NS	NS	NS	NS	NS	NS	NS	NS	NS	NS	NS	NS	NS	NS	NS	NS	NS	NS
P8A vs PCA	NS	NS	NS	NS	NS	NS	NS	NS	NS	NS	NS	NS	NS	NS	NS	NS	NS	NS	NS	NS	NS	NS	NS	NS
*Contrasts to test ‘ontogeny’ effect*^4^																								
J vs A	NS	*	NS	NS	**	NS	NS	**	NS	NS	**	NS	NS	NS	NS	***	***	***	NS	NS	NS	**	**	***
PCJ vs PCA	NS	*	NS	NS	*	NS	NS	*	NS	NS	**	NS	NS	NS	NS	***	*	***	NS	NS	NS	**	NS	***
TJ vs TA	NS	NS	NS	NS	NS	NS	NS	*	NS	NS	*	NS	NS	NS	NS	*	***	***	NS	NS	NS	NS	**	***

^1^The “juice content” variable was log-transformed prior to the analyses to fit the data to a normal distribution.

^2^ANOVA to test the effects of Line on each fruit quality variable. Independent statistical analyses were performed for each fruit quality parameter and season.

^3^Contrasts to test for significant differences between each adult transgenic line and their respective control line (PCA) using Dunnett’s test.

^4^Planned comparisons to test for significant differences between juvenile and adult lines. J, average of all juvenile lines; A, average of all adult lines; TJ, average of all juvenile and transgenic lines; TA, average of all adult and transgenic lines.

TA, titratable acidity; TSS, total soluble solids; MI, maturity index (TSS/TA); S1, season 2004; S2, season 2005; S3, season 2006.

*p < 0.01; **p < 0.001; ***p < 0.0001; NS, not significant.

A summary of the fruit quality characteristics of the citrange lines is presented in Additional file 2. There were no noticeable differences in yield among the seasons analyzed. The effects of ‘line’, ‘transgene’ and ‘ploidy’ on the fruit quality of the citrange lines are presented in Table 3. The ANOVA results showed that the effect of ‘line’ on all of the fruit quality variables was significant for at least two of the four seasons analyzed, at p < 0.01, with the exception of the variable CI. Regarding the ‘transgene’ effect, Table 3 also shows that no significant differences were found for more than one season for any of the quality parameters evaluated when each transgenic line was compared with its corresponding non-GM control. For most of the variables (V, W, caliber and JC), significant differences were found exclusively for the first season analyzed, and these differences decreased in the following seasons, ceasing to be significant (p < 0.01) in all cases. This result may indicate that the citrange trees were not fully mature in the first year of assessment (S1). Thus, it was necessary to evaluate these parameters in an additional (fourth) year (S4) to confirm that the highly significant differences found for S1 were not repeated and, therefore, could not be attributed to the ‘transgene’ effect. Table 3 shows that ‘ploidy’ had a significant effect on W, V, caliber and JC in all of the four seasons analyzed. Moreover, for these variables, the differences between the diploid (T-2n) and tetraploid (T-4n) transgenic lines were highly significant (p < 0.001) for at least two of the four seasons. ‘Ploidy’ also had a significant effect on the MI in S1, S2 and S3, although at a lower significance level than the other variables tested (Figure 6). The tetraploid lines showed higher W, V, and caliber and lower JC values than the diploid lines (Figure 6B), indicating that the higher weight and size of the tetraploid fruit were due to a greater peel thickness and not to a higher juice percentage (Figure 6C). For these variables, the trend of the compared means within the contrasts was consistent over the seasons, meaning that these differences between the diploid and tetraploid lines, in addition to being highly significant, were consistent, regardless of the season/environmental conditions. The tetraploid lines also showed higher MI values than the diploid lines. This less-pronounced but consistent ‘ploidy’ effect was due to a lower TA in the tetraploid lines compared to the diploid lines.

**Table 3:** The effects of the ‘line’, ‘transgene’ and ‘ploidy’ factors on the fruit quality in the citrange lines

	**Fruit quality parameter (dependent variable)**																							
**Source**	**Weight**				**Volume**				**Caliber**				**Color Index**				**Juice Content**^**1**^				**MI (TSS/TA)**			
	**S1**	**S2**	**S3**	**S4**	**S1**	**S2**	**S3**	**S4**	**S1**	**S2**	**S3**	**S4**	**S1**	**S2**	**S3**	**S4**	**S1**	**S2**	**S3**	**S4**	**S1**	**S2**	**S3**	**S4**
*Factor /ANOVA*^*2*^																								
Line	***	*	**	NS	***	*	***	NS	***	*	***	NS	NS	NS	NS	–	**	***	*	-	*	*	NS	-
Plant(Line)	***	***	***	***	***	***	***	***	***	***	***	***	***	***	***	–	*	NS	***	-	***	***	*	-
*Contrasts to test ‘transgene’ effect*^*3*^																								
C1 vs CC	***	NS	NS	NS	***	NS	NS	NS	***	NS	NS	NS	NS	NS	NS	–	*	NS	NS	–	NS	NS	NS	–
C3 vs CC	*	NS	NS	NS	NS	NS	NS	NS	*	NS	NS	NS	NS	NS	NS	–	*	NS	NS	–	NS	NS	NS	–
C4 vs CC	NS	NS	NS	NS	NS	NS	NS	NS	NS	NS	NS	NS	NS	NS	NS	–	NS	NS	NS	–	NS	*	NS	–
C5 vs CC	**	NS	NS	NS	**	NS	NS	NS	**	NS	NS	NS	NS	NS	NS	–	NS	NS	*	–	NS	NS	NS	–
C6 vs CC	**	NS	NS	NS	*	NS	NS	NS	*	NS	NS	NS	NS	NS	NS	–	*	NS	NS	–	NS	NS	NS	–
C8 vs CC	NS	NS	NS	NS	NS	NS	NS	NS	NS	NS	NS	NS	NS	NS	NS	–	NS	NS	NS	–	NS	NS	NS	–
*Contrasts to test ‘ploidy’ effect*^*4*^																								
T-2n vs T-4n	***	***	***	*	***	***	***	**	***	***	***	**	NS	NS	NS	–	*	***	**	-	*	**	*	-

^1^The “juice content” variable was transformed prior to the analyses to fit the data to a normal distribution.

^2^ANOVA to test the effects of Line on each fruit quality variable. Independent statistical analyses were performed for each fruit quality parameter and season.

^3^Contrasts to test for significant differences between each transgenic diploid line and their respective control line (CC) using Dunnett’s test. In the absence of a tetraploid control line, transgenic lines C2 and C7 (tetraploids) were excluded from this analysis to avoid confounding effects.

^4^Planned comparisons to test for significant differences between the average of all transgenic diploid lines (T-2n) and the average of all transgenic tetraploid lines (T-4n). Because only one control line (CC) was available for the Carrizo citrange plants, which was diploid, the data from this line were excluded from this analysis to avoid confounding effects.

MI, maturity index; TSS, total soluble solids; TA, titratable acidity; S1, season 2004; S2, season 2005; S3, season 2006; S4, season 2007.

-, Not measured; *p < 0.01; **p < 0.001; ***p < 0.0001; NS, not significant.

In summary, the results from the analysis of fruit quality indicated that (1) no significant ‘transgene’ effect was detected for any fruit quality parameter evaluated, and (2) both the methods of evaluation and the statistical analyses performed to study the influence of transgenesis on the fruit quality of the different citrus genotypes were robust and sufficiently powerful to detect differences due to other physiological and genetic factors.

## Discussions

For such long-lived and vegetatively propagated crops with complex genetic and reproductive characteristics as fruit trees, genetic modification offers an important potential for crop improvement. Genetic engineering allows desirable traits to be transferred into mature tissues of selected genotypes, bypassing the long crossing cycles required in tree breeding programs. Moreover, genetic engineering overrides incompatibility barriers and permits gene transfer not only between unrelated tree species, but also between widely divergent taxa. Additionally, the potentially undesirable effects of linked alleles, which could be inadvertently introduced into the progeny in conventional breeding programs, can be avoided. However, the future prospects for commercial plantations of GM trees are controversial and remain uncertain, as certain biological and regulatory issues still need to be satisfactorily resolved [42-46]. The modification of crops *via* genetic engineering is a subject of public concern. A question that is often asked is “do genetically modified crops differ significantly from their non-modified equivalents?” The term ‘substantial equivalence’ has been used in the fields of food safety and biotechnology to describe the relationship between components produced from the same source using either novel or conventional methodologies: if the resulting components are indistinguishable, they can be considered equivalent [47]. Substantial equivalence in the context of this work is used to describe the relationship between the phenotype and agronomic performance of the GM citrus plants and their non-GM counterparts.

We report here that several independent transgenic sweet orange and citrange lines stably carrying and expressing *uidA* and *nptII* transgenes showed a similar phenotype (at morphological, phenological and agronomic levels) to their non-transgenic comparators when both were grown under orchard conditions for a long period of time (> 7 years). We intentionally used transgenes with a well-characterized function to simplify the analysis of substantial equivalence and because this was the first release of transgenic citrus plants into the field. The evaluated parameters allowed the assessment of the outcomes of numerous metabolic pathways that would tentatively result in a distinguishable phenotype in the modified plants, as recommended in the Guidance Document (Section III, D7) described by the EFSA GMO Panel [29]. Moreover, some aspects regarding the design of the experimental orchard contributed to the validation of our study. The relatively high number of independent transgenic lines (eight) of each citrus type used allowed the minimization of event-specific unintended effects derived from transgene integration. The availability of more than one plant per line permitted investigating the intraline variability and discarding possible chimeric events, which frequently occur during the genetic transformation of citrus [48]. The homogeneous distribution of the non-transgenic control trees within the orchard contributed to reducing the possible environmental effects caused by the position of the trees in the field. Lastly, the inclusion of some off-type lines from each citrus type (juvenile sweet orange and tetraploid citrange lines) allowed assessing the influence of other (genetic and physiological) factors on the parameters studied. Thus, by performing the comparisons “juvenile versus adult” for the sweet orange lines and “diploid versus tetraploid” for the citrange lines, we also addressed the effects of ‘ontogeny’ and ‘ploidy’ on the phenotypic variables.

There are reported cases of transgenic trees in which the expression of transgenes was silenced at some point during development [34,49]. There are also instances of T-DNA loss, such as in transgenic apples [50], which are likely due to chimerism rather than T-DNA instability. In general, a high stability of transgene integration and expression have been observed in trees over 3 to 4 years of culture *in vitro* in either the greenhouse or in the field [51,52]. However, there is limited information available about the stability of transgene expression over the many years that trees remain in the field, where they are subjected to highly variable environmental conditions. The results of our molecular analyses confirmed the long-term stability of transgene insertion and expression over 7 years for all of the transgenic citrus lines examined. Moreover, little seasonal variation in the expression levels was detected between plants of the same transgenic line in different organs and over the duration of the study, confirming the absence of rearrangements and/or silencing of the transgenes after transferring the plants to the orchard conditions. The long-term stability of *attacin E* transgene expression has also been recently shown in orchard-grown apples trees over a 12-year period [53].

The monitoring of commercial transgenic crop varieties in the field has allowed the observation of unintended traits. Verified examples of such traits include stem splitting and decreased yields in transgenic soybean plants [54] and a 67-fold reduction in beta-carotene content in a transgenic squash variety engineered for virus resistance (USDA Application # 95-352-01). Therefore, it is important to test whether the stable expression of transgenes in different organs affects morphological, phenological and fruit quality parameters, especially in perennial crops. While investigating apple, Ruhmann et al. [55] have shown that the expression of a stilbene synthase transgene did not affect the leaf shape, flower morphology and color, or fruit shape and size when compared to control plants and fruit. *Attacin E* overexpression also did not affect the fruit characteristics of transgenic apple fruit of trees grown in the field over a period of 7 years [53]. No significant differences in the morphological variables or fruit quality parameters have been found between the transgenic and non-transformed controls of the two citrus genotypes tested in our study. Furthermore, the evaluation methods and statistical analyses used for this purpose were robust and sufficiently powerful to detect significant differences when comparing trees at different developmental stages (for sweet orange) or with different ploidy levels (for citrange). These results indicated that the modification of the citrus genome via conventional breeding (with the subsequent generation of -juvenile- seedlings) or *via* ploidy manipulation (i.e., through polyploidization processes) generates much more genetic and phenotypic variability in terms of morphology and fruit quality than is induced by genetic engineering. Therefore, transgenesis can be considered to be a more precise method for altering genotypes, without (or minimally) affecting phenotypes in comparison with other breeding methods commonly used in citriculture.

The goal of genetic engineering in crop improvement programs generally involves the modification of metabolic pathways in a manner that may alter plant development and/or fitness under real agricultural conditions at much more complex levels than those described here. However, particular attention should be paid to the selectable marker genes used, as they usually remain linked to the transgenes of interest, at least in vegetatively propagated crops. The detailed pleiotropic effects of selectable marker genes need to be understood, as they may influence the interpretation of scientific results when co-transforming genes of interest are being examined in transgenic plants [3]. Our research has shown that *nptII* and *uidA* did not induce pleiotropic effects on the main phenotypic plant characteristics of transgenic citrus trees.

## Conclusions

We have demonstrated that the stable integration and expression of *uid*A and *nptII* transgenes for more than 7 years under orchard conditions has minimal effects on the main agronomic plant characteristics (tree morphology, phenology and fruit quality) of transgenic citrus lines compared to appropriate controls. Therefore, transgenic sweet orange and citrange lines carrying the selectable marker genes that are most commonly used in citrus transformation are substantially equivalent to the non-transformed controls during long-term agricultural cultivation. This information is essential to be able to focus mainly on the pleiotropic effects that may be induced by the insertion of gene(s) of interest in future experiments with GM citrus.

## Methods

### Plant materials and experimental field design

The citrus transformants and controls used in this work (see Figure 1A) were generated previously in our laboratory. *A. tumefaciens* EHA 105 containing the binary plasmid p35SGUSINT was used in the different experiments as a vector for the transformation of plant materials from three citrus types: Pineapple sweet orange (*C. sinensis* L. Osb.) [35]; Carrizo citrange (*Citrus sinensis* L. Osb. X *Poncirus trifoliata* L. Raf.) [37,38] and Mexican lime (*C. aurantifolia* (Christm.) Swing.) [39]. Two gene cassettes in the T-DNA, 35 S-*uidA*(GUSINT)-35 S and NOS-*nptII*-NOS, served as the reporter and selectable marker genes, respectively. Six independent sweet orange transgenic lines derived from adult plant material (designated P3 to P8) and two derived from juvenile material (designated P1 and P2) were selected for the release. Non-GM regenerants obtained from these transformation experiments served as the control adult (PCA) and juvenile (PCJ) sweet orange lines. For the release, we also selected six independent transgenic citrange lines (designated C1, C3, C4, C5, C6 and C8) and one non-GM regenerant, which was used as a control line (CC). Moreover, we included two off-type transgenic tetraploid lines (designated C2 and C7) in the orchard that were unintentionally obtained during the course of the experiments [33]. The Mexican lime plants included in the field trial (named L1 to L8 and LC) were excluded from the study because they suffered severe symptoms from frost in several consecutive winters.

The transgenic lines were chosen based on their high level of transgene expression and low copy number of transgene insertions. The plants were transferred to the orchard conditions in 1997, together with their respective non-GM controls. The experimental orchard, designated the T plot, was located at the Instituto Valenciano de Investigaciones Agrarias, Spain (latitude 39°35”N, longitude 0°23”W and elevation of 50 m; typical Mediterranean climate), and was approved by the Spanish Ministry of Environment (permit Nr. B/ES/96/15). All of the scion types were grafted onto Carrizo citrange rootstock and grown in a loamy clay soil using drip irrigation. The orchard was managed as for normal citrus cultivation. The T plot, which covered an area of 1.638 m^2^, contained 130 trees distributed in rows, as described in Figure 1C. Non-transgenic Clemenules clementine (*C. clementina* ex. Hort. Tan.) trees planted along an external edge were used as a buffer to prevent transgene flow through pollen dispersal [56]. It was designed to study long-term transgene integration/expression and the influence of transgenesis (the ‘transgene’ effect) on the main phenotypic plant characteristics.

### Molecular characterization

#### Southern blot analysis

Genomic DNA was isolated from leaves according to Dellaporta et al. (1983) [57]. The Southern blot analysis was performed using 20 μg of *Eco*RI-, *Dra*I-, *Hin*dIII- and *Dra*I + *Cla*I-digested samples, which were separated on 1 % (w/v) agarose gels and blotted onto nylon membranes (Hybond-N+, Amersham,, Buckinghamshire, UK). The filters were probed with a digoxigenin (Boehringer-Mannheim, East Sussex, UK)-labeled fragment corresponding to the coding region of the *uidA* or the *nptII* gene prepared by PCR following the supplier’s instructions.

#### Histochemical and fluorimetric GUS assays and NPTII ELISA

The histochemical GUS activity of the transgenic plants was analyzed as described in [37]. The GUS activity in the leaf samples was estimated by measuring the fluorescence emitted at 445 nm during the hydrolysis of 4-MUG to 4-MU [58]. The NPTII activity in leaf samples was quantitated using a commercial Patho Screen NPTII ELISA kit (Agdia Inc., Indiana, USA). The GUS and NPTII analyses were performed using crude protein samples extracted from the fully expanded leaves from each plant. The total protein was quantified using the Bradford assay.

### Phenotypic characterization

#### Morphology

To analyze the size of the trees, measurements of the height and average diameter for each tree were recorded at the end of the growing season. We defined the tree height (TH) as the highest point of the plant measured from the soil. The average diameter was calculated from two independent measurements of the diameter of the tree obtained at different points. The tree canopy volume (TCV) was calculated by applying the volume formula for the ellipsoid, as follows: V = 0.524 h d^2^, where “h” is the TH and “d” is the average diameter of the tree. To study leaf morphology, the average leaf fresh weight (LFW) and average leaf area (LA) parameters were calculated for each tree. Measurements were performed using 30 adult leaves located in the intermediate zone of spring shoots. The area was measured using a LiCor 3100 C device (Nebraska, USA).

Statistical analyses were performed using STATGRAPHICS Plus software, version 5.0. Each citrus genotype was analyzed separately. The data for each morphological variable (TH, TCV, LFW and LA) were analyzed using the Kruskal-Wallis non-parametric test to determine whether differences in the median values existed among the lines [59]. The effects of the ‘transgene’, ‘ontogeny’ and ‘ploidy’ factors were tested by performing pertinent planned comparisons using the Mann–Whitney non-parametric test. We chose these tests because the data did not show clear normality or equal variances. Moreover, Kruskal-Wallis is a recommended as an alternative to parametric analysis of variance (ANOVA) for populations containing uneven sample sizes [60], as was the case in the present study. The Kruskal-Wallis test compares the medians instead of the means; therefore, we report the medians and interquartile ranges instead of the means and standard deviations for these variables.

#### Phenology

The phenological cycle of every tree in the orchard was evaluated through weekly observations and the recording of the predominant phenological stage of development according to BBCH codifications [61]. A visual representation of the phenological cycle of each line was produced by generating phenological calenders.

#### Analysis of fruit quality

The assessment of fruit quality for the sweet orange and citrange lines was performed for 3 and 4 consecutive seasons, respectively, starting in the 2004 production season in both cases. Measurements of quality parameters were performed based on fruit samples from every citrus tree in the T plot. A total of 30 fruits (six samples of 5 fruits each) per tree were harvested annually when the fruit was fully mature. The following fruit quality parameters were measured and averaged for each sample: fruit weight (W), fruit volume (V), caliber, the color index (CI), juice content (JC), total soluble solids (TSS), titratable acidity (TA) and maturity index (MI). The V was estimated *via* the water displacement method. To estimate the caliber, the equatorial diameter of the fruit was measured using MITUTOYO digital calipers (Ilinois, USA). The CI was determined according to the method described by Jiménez-Cuesta et al. (1981) [62]. The L (0-100, black to white), a (± red/green) and b (±yellow/blue) parameters of the color system were measured using a Minolta CR-200 Chroma Meter (Osaka, Japan). The juice was extracted from the fruit and weighed, and the JC was expressed as a percentage on the basis of weight. Immediately after the extraction of the juice, the TSS was determined in terms of Brix degrees using a refractometer (Atago PR-101 model 0-45 %, Tokyo, Japan). The TA of the juice was determined by titration with 0.1 mol L^-1^ NaOH and expressed as the percentage of anhydrous citric acid by weight, using phenolphthalein as a visual endpoint indicator, according to AOAC methods (AOAC. 1980. Official Methods of Analysis, 13th ed. N°46024 and N° 22061. Association of Official Analytical Chemists, Washington. DC). The MI was estimated as the TSS/TA ratio.

Prior to the statistical analysis, the quality variables were checked for normality, and those that deviated were transformed *via* log transformation. A double hierarchical analysis of variance was conducted using the General Linear Models procedure (GLM, for ANOVA with unbalanced data) to assess the influence of ‘line’ (independent variable) on the variance of each fruit quality parameter measured (dependent variable). The analysis was performed separately for each citrus type and season, and the model used was as follows: x_ij_= μ+ line_i_ + plant (line)_j(i)_ + error_k(ij)_. The main factor, ‘line’, included the C1, C2, C3, C4, C5, C6, C7, C8, and CC treatments for the citrange samples and the P1, P2, P3, P4, P5, P6, P7, P8, PCJ, and PCA treatments for the sweet orange samples. The hierarchical factor, ‘plant’, included the plant treatments within each level of ‘line’. The ‘plant’ effect was considered random, and it was used as the source of error for the ‘line’ effect. We used the restricted maximum-likelihood estimation technique to avoid negative estimates of variance. *A posteriori*, we used Dunnett’s test to address the effect of ‘transgene’ (each transgenic line vs. control) on each fruit quality variable. Additionally, the effects of ‘ploidy’ (2n vs. 4n) and ‘ontogeny’ (juvenile vs. adult) were also addressed in the citrange and sweet orange lines, respectively, by performing the corresponding planned (or *a priori*) contrasts/comparisons. The statistical analyses were all performed using the software package SAS version 8.02 (SAS Institute Inc., Cary, NC, USA), and a significance level (α) of 0.01 was taken into consideration to protect against Type I errors.

## Abbreviations

CaMV, Cauliflower mosaic virus; 4-MUG, 4-methyl-umbelliferyl-β-D-glucuronide; 4-MU, 4-methylumbelliferone; CI, Color index; DNA, Deoxyribonucleic acid; EFSA, European Food Safety Authority; ELISA, Enzyme Linked Immunosorbent Assay; GM, Genetic modification, or genetically modified; GMO, Genetically modified organism; GUS, β-glucuronidase; GUSINT, GUS intron; JC, Juice content; LA, Average leaf area; LFW, Leaf fresh weight; MI, Maturity index; NOS, Nopaline synthase; *nptII*, Neomycin phosphotransferase II gene; PCR, Polymerase chain reaction; TA, Titratable acidity; TCV, Tree canopy volume; T-DNA, Transfer DNA; TH, Tree height; TSS, Total soluble solids; *Uid*, β-glucuronidase gene; V, Fruit volume; W, Fruit weight.

## Competing interests

The authors declare that they have no competing interests.

## Authors’ contributions

EP performed the molecular and phenotypic analyses of the transgenic trees, analyzed the data and drafted the manuscript. JEP performed the phenotypic analysis. LP conceived and designed the experiments, contributed to analyzing the data and drafted the manuscript. All of the authors read and approved the final manuscript.

## Supplementary Material

Additional file 1 Summary of the analysis of fruit quality for the transgenic sweet orange lines. Click here for file

Additional file 2 Summary of the analysis of fruit quality for the transgenic citrange lines. Click here for file
