# Supplementary material for: Field performance of transgenic citrus trees: Assessment of the long-term expression of uidA and nptII transgenes and its impact on relevant agronomic and phenotypic characteristics
Source: BMC Biotechnol. 2012 Jul 15;12:41. doi: 10.1186/1472-6750-12-41 (PMC3462728; doi:10.1186/1472-6750-12-41)
Supplement: Additional file 1 — Summary of the analysis of fruit quality for the transgenic sweet orange lines. [file 1472-6750-12-41-S1.doc]

**Additional file 1. Summary of the analysis of fruit quality for all sweet orange lines**.

| **Season** | **Line** | **Sampling** | | | |  | **Fruit quality parameter** | | | | | | | |
| --- | --- | --- | --- | --- | --- | --- | --- | --- | --- | --- | --- | --- | --- | --- |
|  |  | trees/  line | samples/tree | *n*1 | *n*2 |  | Weight (g) | Volume (ml) | Caliber (mm) | Color Index | JC (%) | TSS (%) | TA (%) | MI (TSS/TA) |
| S1 (2004) | PCJ | 2 | 6 | 12 | 12 |  | 149.83 ± 3.08 | 182.25 ± 3.89 | 68.74 ± 0.53 | 12.23 ± 0.27 | 45.59 ± 0.92 | 13.09 ± 0.30 | 1.34 ± 0.03 | 9.77 ± 0.23 |
|  | P1 | 2 | 6 | 12 | 12 |  | 149.18 ± 5.03 | 171.18 ± 6.87 | 68.14 ± 0.90 | 14.88 ± 0.24 | 43.47 ± 1.19 | 13.13 ± 0.24 | 1.32 ± 0.01 | 9.98 ± 0.18 |
|  | P2 | 2 | 6 | 12 | 12 |  | 119.50 ± 2.14 | 138.75 ± 3.28 | 62.91 ± 0.38 | 11.98 ± 0.17 | 40.12 ± 0.46 | 12.80 ± 0.10 | 0.79 ± 0.02 | 16.25 ± 0.40 |
|  | PCA | 6 | 6 | 36 | 36 |  | 150.22 ± 4.17 | 170.33 ± 5.24 | 67.72 ± 0.63 | 14.10 ± 0.25 | 42.10 ± 0.52 | 13.39 ± 0.08 | 0.81 ± 0.02 | 16.86 ± 0.42 |
|  | P3 | 2 | 6 | 12 | 12 |  | 155.33 ± 5.21 | 178.33 ± 6.08 | 69.39 ± 0.83 | 14.34 ± 0.39 | 46.44 ± 0.83 | 13.33 ± 0.13 | 0.87 ± 0.02 | 15.57 ± 0.52 |
|  | P4 | 2 | 6 | 12 | 12 |  | 154.08 ± 3.06 | 177.17 ± 4.17 | 69.16 ± 0.52 | 15.23 ± 0.19 | 41.61 ± 0.78 | 12.90 ± 0.07 | 0.97 ± 0.01 | 13.28 ± 0.17 |
|  | P5 | 2 | 6 | 12 | 12 |  | 121.92 ± 3.16 | 136.00 ± 3.43 | 61.90 ± 0.57 | 13.11 ± 0.35 | 47.04 ± 0.53 | 13.87 ± 0.09 | 0.86 ± 0.02 | 16.21 ± 0.39 |
|  | P6 | 2 | 6 | 12 | 12 |  | 128.42 ± 2.26 | 139.67 ± 3.86 | 63.49 ± 0.41 | 12.96 ± 0.16 | 43.13 ± 0.70 | 13.38 ± 0.09 | 0.94 ± 0.02 | 14.33 ± 0.25 |
|  | P7 | 2 | 6 | 12 | 12 |  | 137.00 ± 3.21 | 149.83 ± 3.41 | 65.76 ± 0.60 | 14.62 ± 0.21 | 41.88 ± 0.42 | 13.10 ± 0.09 | 0.91 ± 0.01 | 14.44 ± 0.18 |
|  | P8 | 2 | 6 | 12 | 12 |  | 144.58 ± 2.43 | 155.17 ± 2.78 | 66.61 ± 0.40 | 13.26 ± 0.21 | 44.05 ± 0.79 | 13.44 ± 0.10 | 0.81 ± 0.03 | 16.82 ± 0.63 |
| S2 (2005) | PCJ | 2 | 6 | 12 | **8** |  | 239.38 ± 20.75 | 284.75 ± 40.11 | 80.60 ± 2.75 | 10.59 ± 0.16 | 41.76 ± 0.90 | 11.90 ± 0.32 | 1.15 ± 0.05 | 10.46 ± 0.45 |
|  | P1 | 2 | 6 | 12 | **8** |  | 230.00 ± 4.19 | 297.25 ± 10.45 | 81.62 ± 0.58 | 12.60 ± 0.18 | 43.84 ± 1.06 | 12.66 ± 0.13 | 1.43 ± 0.02 | 8.87 ± 0.18 |
|  | P2 | 2 | 6 | 12 | **8** |  | 166.88 ± 6.76 | 193.63 ± 18.14 | 68.73 ± 1.43 | 10.14 ± 0.32 | 39.67 ± 0.83 | 12.84 ± 0.27 | 0.99 ± 0.01 | 13.00 ± 0.28 |
|  | PCA | 6 | 6 | 36 | 36 |  | 147.67 ± 4.67 | 162.61 ± 5.58 | 67.44 ± 0.70 | 14.02 ± 0.19 | 41.36 ± 0.67 | 12.01 ± 0.06 | 0.92 ± 0.01 | 13.12 ± 0.18 |
|  | P3 | 2 | 6 | 12 | 12 |  | 150.42 ± 9.53 | 161.00 ± 9.86 | 64.82 ± 1.90 | 12.43 ± 0.15 | 41.81 ± 0.49 | 12.09 ± 0.11 | 0.95 ± 0.03 | 12.82 ± 0.38 |
|  | P4 | 2 | 6 | 12 | 12 |  | 166.42 ± 14.17 | 186.08 ± 16.90 | 70.17 ± 2.07 | 13.01 ± 0.43 | 42.05 ± 0.89 | 12.13 ± 0.06 | 0.98 ± 0.01 | 12.42 ± 0.20 |
|  | P5 | 2 | 6 | 12 | 12 |  | 120.00 ± 2.51 | 127.17 ± 2.64 | 61.78 ± 0.52 | 12.90 ± 0.28 | 44.81 ± 0.67 | 12.78 ± 0.09 | 0.82 ± 0.03 | 15.84 ± 0.55 |
|  | P6 | 2 | 6 | 12 | 12 |  | 154.83 ± 3.96 | 167.17 ± 3.75 | 68.43 ± 0.63 | 13.76 ± 0.32 | 41.10 ± 0.72 | 12.37 ± 0.20 | 0.88 ± 0.02 | 14.01 ± 0.20 |
|  | P7 | 2 | 6 | 12 | 12 |  | 125.33 ± 5.20 | 134.17 ± 5.57 | 63.06 ± 0.97 | 14.43 ± 0.15 | 40.47 ± 0.83 | 12.50 ± 0.09 | 0.93 ± 0.01 | 13.49 ± 0.17 |
|  | P8 | 2 | 6 | 12 | 12 |  | 144.42 ± 4.84 | 156.50 ± 5.61 | 66.45 ± 0.85 | 12.81 ± 0.32 | 41.34 ± 0.91 | 12.54 ± 0.21 | 0.87 ± 0.01 | 14.53 ± 0.36 |
| S3 (2006) | PCJ | 2 | 6 | 12 | 12 |  | 176.58 ± 3.95 | 199.00 ± 3.94 | 73.11 ± 0.64 | 12.36 ± 0.53 | 46.68 ± 0.43 | 12.13 ± 0.09 | 1.46 ± 0.02 | 8.33 ± 0.10 |
|  | P1 | 2 | 6 | 12 | 12 |  | 197.50 ± 10.49 | 221.17 ± 11.91 | 74.16 ± 1.24 | 13.71 ± 0.25 | 44.97 ± 0.64 | 12.15 ± 0.14 | 1.53 ± 0.03 | 7.97 ± 0.18 |
|  | P2 | 2 | 6 | 12 | 12 |  | 144.67 ± 2.65 | 161.17 ± 3.24 | 67.08 ± 0.52 | 11.89 ± 0.40 | 45.64 ± 0.33 | 12.29 ± 0.22 | 1.00 ± 0.03 | 12.33 ± 0.21 |
|  | PCA | 6 | 6 | 36 | **24** |  | 216.54 ± 3.85 | 242.63 ± 4.68 | 76.22 ± 0.45 | 13.15 ± 0.17 | 44.39 ± 0.70 | 11.91 ± 0.11 | 0.85 ± 0.01 | 14.03 ± 0.16 |
|  | P3 | 2 | 6 | 12 | **6** |  | 196.00 ± 2.56 | 228.33 ± 5.76 | 73.97 ± 0.47 | 13.79 ± 0.34 | 46.63 ± 0.78 | 11.47 ± 0.19 | 0.99 ± 0.03 | 11.68 ± 0.32 |
|  | P4 | 2 | 6 | 12 | **8** |  | 237.75 ± 14.74 | 277.13 ± 18.83 | 79.27 ± 1.50 | 12.35 ± 0.23 | 43.35 ± 0.66 | 11.40 ± 0.20 | 1.05 ± 0.03 | 10.86 ± 0.18 |
|  | P5 | 2 | 6 | 12 | 12 |  | 159.58 ± 3.91 | 173.67 ± 5.08 | 68.48 ± 0.63 | 11.58 ± 0.31 | 45.78 ± 0.64 | 11.94 ± 0.07 | 0.88 ± 0.01 | 13.60 ± 0.23 |
|  | P6 | 2 | 6 | 12 | 12 |  | 170.25 ± 3.43 | 193.58 ± 4.03 | 71.15 ± 0.54 | 13.59 ± 0.36 | 43.16 ± 0.46 | 11.88 ± 0.07 | 0.94 ± 0.02 | 12.70 ± 0.27 |
|  | P7 | 2 | 6 | 12 | 12 |  | 176.50 ± 2.11 | 199.83 ± 2.40 | 72.18 ± 0.44 | 13.01 ± 0.35 | 44.73 ± 0.61 | 11.27 ± 0.11 | 0.90 ± 0.01 | 12.49 ± 0.13 |
|  | P8 | 2 | 6 | 12 | 12 |  | 177.25 ± 5.43 | 197.92 ± 6.70 | 71.72 ± 0.73 | 11.85 ± 0.40 | 44.23 ± 0.67 | 11.85 ± 0.09 | 0.88 ± 0.02 | 13.44 ± 0.19 |

*n*1, theorical/planned sampling; *n*2, sampling carried out. The cases where yield was very scarce (*n*2 < *n*1) are shown in bold.

Each value represents the average ± SE of the *n2* samples analyzed per line and year.

JC, juice content; TSS, total soluble solids; TA, titratable acidity; MI, maturity index
