# Supplementary material for: Field performance of transgenic citrus trees: Assessment of the long-term expression of uidA and nptII transgenes and its impact on relevant agronomic and phenotypic characteristics
Source: BMC Biotechnol. 2012 Jul 15;12:41. doi: 10.1186/1472-6750-12-41 (PMC3462728; doi:10.1186/1472-6750-12-41)
Supplement: Additional file 2 — Summary of the analysis of fruit quality for the transgenic citrange lines. [file 1472-6750-12-41-S2.doc]

**Additional file 2. Summary of the analysis of fruit quality for the citrange lines**. Data are the average ± SE of the *n* samples analyzed per line and year. -, Not measured

| **Season** | **Line** | **Sampling** | | |  | **Fruit quality parameter** | | | | | | | |
| --- | --- | --- | --- | --- | --- | --- | --- | --- | --- | --- | --- | --- | --- |
|  |  | trees/ line | samples/  tree | *n* |  | Weight (g) | Volume (ml) | Caliber (mm) | Color Index | JC (%) | TSS (%) | TA (%) | MI (TSS/TA) |
| S1 (2004) | CC | 8 | 6 | 48 |  | 82.63 ± 1.03 | 96.67 ± 1.54 | 54.53 ± 0.29 | 8.61 ± 0.16 | 29.75 ± 0.54 | 11.10 ± 0.09 | 5.26 ± 0.05 | 2.12 ± 0.02 |
|  | C1 | 2 | 6 | 12 |  | 46.75 ± 1.97 | 56.50 ± 3.20 | 45.06 ± 0.73 | 8.16 ± 0.20 | 24.29 ± 1.15 | 12.27 ± 0.15 | 5.22 ± 0.11 | 2.36 ± 0.07 |
|  | C2 | 2 | 6 | 12 |  | 101.63 ± 4.78 | 126.50 ± 4.63 | 59.45 ± 0.92 | 8.13 ± 0.72 | 22.28 ± 1.61 | 11.79 ± 0.23 | 4.91 ± 0.24 | 2.43 ± 0.10 |
|  | C3 | 2 | 6 | 12 |  | 64.75 ± 2.35 | 77.50 ± 3.05 | 49.54 ± 0.46 | 7.73 ± 0.27 | 23.54 ± 1.27 | 11.69 ± 0.22 | 5.11 ± 0.11 | 2.30 ± 0.05 |
|  | C4 | 2 | 6 | 12 |  | 76.42 ± 2.32 | 86.58 ± 3.29 | 53.04 ± 0.55 | 8.28 ± 0.17 | 26.44 ± 0.68 | 12.13 ± 0.20 | 5.69 ± 0.06 | 2.13 ± 0.04 |
|  | C5 | 2 | 6 | 12 |  | 60.58 ± 2.15 | 67.83 ± 2.99 | 48.24 ± 0.66 | 8.82 ± 0.44 | 34.06 ± 1.30 | 10.70 ± 0.19 | 5.19 ± 0.09 | 2.07 ± 0.03 |
|  | C6 | 2 | 6 | 12 |  | 60.08 ± 2.04 | 72.17 ± 2.98 | 49.76 ± 0.55 | 8.26 ± 0.23 | 23.71 ± 1.48 | 11.69 ± 0.15 | 5.55 ± 0.12 | 2.11 ± 0.04 |
|  | C7 | 2 | 6 | 12 |  | 84.92 ± 2.39 | 104.83 ± 3.58 | 57.75 ± 0.66 | 9.27 ± 0.26 | 20.84 ± 0.69 | 11.78 ± 0.12 | 4.63 ± 0.08 | 2.56 ± 0.05 |
|  | C8 | 2 | 6 | 12 |  | 78.17 ± 1.77 | 93.00 ± 2.21 | 54.36 ± 0.43 | 9.76 ± 0.28 | 29.96 ± 1.22 | 11.28 ± 0.14 | 4.92 ± 0.07 | 2.30 ± 0.04 |
| S2 (2005) | CC | 8 | 6 | 48 |  | 83.46 ± 1.79 | 96.58 ± 2.16 | 55.70 ± 0.44 | 2.08 ± 1.24 | 33.82 ± 0.35 | 11.83 ± 0.12 | 5.61 ± 0.08 | 2.12 ± 0.02 |
|  | C1 | 2 | 6 | 12 |  | 60.75 ± 1.88 | 74.75 ± 4.35 | 49.63 ± 0.57 | -3.64 ± 1.37 | 31.75 ± 1.23 | 11.60 ± 0.17 | 5.34 ± 0.07 | 2.18 ± 0.04 |
|  | C2 | 2 | 6 | 12 |  | 102.67 ± 5.91 | 128.75 ± 7.70 | 62.30 ± 1.31 | -1.82 ± 1.33 | 25.94 ± 1.11 | 11.49 ± 0.08 | 5.16 ± 0.08 | 2.23 ± 0.03 |
|  | C3 | 2 | 6 | 12 |  | 72.00 ± 4.06 | 81.50 ± 4.53 | 52.77 ± 0.94 | 1.57 ± 0.68 | 33.06 ± 0.80 | 11.70 ± 0.13 | 6.14 ± 0.08 | 1.91 ± 0.03 |
|  | C4 | 2 | 6 | 12 |  | 58.27 ± 0.96 | 67.64 ± 1.48 | 49.61 ± 0.33 | 8.57 ± 0.31 | 30.07 ± 0.64 | 13.40 ± 0.06 | 7.12 ± 0.08 | 1.88 ± 0.01 |
|  | C5 | 2 | 6 | 12 |  | 62.17 ± 2.57 | 71.33 ± 2.85 | 50.49 ± 0.72 | 8.35 ± 0.32 | 37.17 ± 0.68 | 12.38 ± 0.06 | 5.99 ± 0.07 | 2.07 ± 0.02 |
|  | C6 | 2 | 6 | 12 |  | 64.42 ± 2.83 | 73.50 ± 2.92 | 51.17 ± 0.76 | 7.84 ± 0.17 | 31.53 ± 0.57 | 13.43 ± 0.15 | 6.20 ± 0.11 | 2.17 ± 0.03 |
|  | C7 | 2 | 6 | 12 |  | 105.08 ± 1.80 | 121.58 ± 1.89 | 61.29 ± 0.39 | 9.27 ± 0.30 | 25.10 ± 0.59 | 12.38 ± 0.13 | 5.15 ± 0.12 | 2.41 ± 0.05 |
|  | C8 | 2 | 6 | 12 |  | 85.58 ± 2.01 | 95.92 ± 2.41 | 56.24 ± 0.49 | 8.60 ± 0.23 | 33.54 ± 0.59 | 11.59 ± 0.08 | 5.36 ± 0.05 | 2.17 ± 0.03 |
| S3 (2006) | CC | 8 | 6 | 48 |  | 77.10 ± 1.24 | 86.71 ± 1.27 | 53.80 ± 0.31 | 4.50 ± 0.17 | 34.46 ± 0.51 | 10.66 ± 0.06 | 5.74 ± 0.08 | 1.88 ± 0.04 |
|  | C1 | 2 | 6 | 12 |  | 72.33 ± 1.97 | 82.00 ± 2.12 | 52.11 ± 0.57 | 4.53 ± 0.48 | 33.96 ± 1.07 | 10.62 ± 0.13 | 5.88 ± 0.11 | 1.81 ± 0.02 |
|  | C2 | 2 | 6 | 12 |  | 141.17 ± 7.93 | 168.08 ± 9.47 | 66.97 ± 1.40 | 2.71 ± 0.51 | 29.98 ± 0.78 | 10.57 ± 0.07 | 5.15 ± 0.08 | 2.06 ± 0.04 |
|  | C3 | 2 | 6 | 12 |  | 78.00 ± 0.96 | 87.58 ± 1.11 | 54.00 ± 0.23 | 3.74 ± 0.25 | 30.67 ± 0.68 | 10.63 ± 0.08 | 5.76 ± 0.05 | 1.85 ± 0.02 |
|  | C4 | 2 | 6 | 12 |  | 74.92 ± 3.42 | 82.50 ± 3.69 | 52.87 ± 0.96 | 4.64 ± 0.20 | 31.81 ± 1.14 | 11.17 ± 0.19 | 5.93 ± 0.12 | 1.89 ± 0.04 |
|  | C5 | 2 | 6 | 12 |  | 59.75 ± 2.35 | 67.00 ± 2.52 | 49.24 ± 0.72 | 5.60 ± 0.35 | 40.42 ± 0.90 | 11.55 ± 0.13 | 5.91 ± 0.10 | 1.96 ± 0.04 |
|  | C6 | 2 | 6 | 12 |  | 63.00 ± 0.99 | 71.33 ± 1.08 | 50.06 ± 0.26 | 4.53 ± 0.47 | 33.32 ± 0.79 | 11.03 ± 0.19 | 5.67 ± 0.12 | 1.95 ± 0.02 |
|  | C7 | 2 | 6 | 12 |  | 100.33 ± 3.03 | 117.67 ± 3.27 | 61.23 ± 0.62 | 5.68 ± 0.26 | 27.82 ± 0.65 | 10.87 ± 0.17 | 5.02 ± 0.06 | 2.17 ± 0.02 |
|  | C8 | 2 | 6 | 12 |  | 71.92 ± 2.07 | 81.33 ± 2.37 | 52.55 ± 0.62 | 4.45 ± 0.41 | 34.66 ± 0.94 | 10.39 ± 0.10 | 5.58 ± 0.08 | 1.87 ± 0.03 |
| S4 (2007) | CC | 8 | 6 | 48 |  | 109.63 ± 2.86 | 123.29 ± 3.38 | 60.62 ± 0.58 | - | - | - | - | - |
|  | C1 | 2 | 6 | 12 |  | 80.33 ± 4.00 | 89.92 ± 4.79 | 54.16 ± 0.90 | - | - | - | - | - |
|  | C2 | 2 | 6 | 12 |  | 133.92 ± 4.72 | 160.50 ± 6.25 | 67.90 ± 0.88 | - | - | - | - | - |
|  | C3 | 2 | 6 | 12 |  | 102.33 ± 4.11 | 116.58 ± 4.70 | 59.58 ± 0.88 | - | - | - | - | - |
|  | C4 | 2 | 6 | 12 |  | 101.17 ± 3.33 | 114.75 ± 3.20 | 59.39 ± 0.62 | - | - | - | - | - |
|  | C5 | 2 | 6 | 12 |  | 75.92 ± 1.83 | 86.42 ± 2.62 | 53.38 ± 0.48 | - | - | - | - | - |
|  | C6 | 2 | 6 | 12 |  | 110.08 ± 5.18 | 119.92 ± 6.03 | 60.25 ± 1.10 | - | - | - | - | - |
|  | C7 | 2 | 6 | 12 |  | 139.00 ± 7.40 | 168.25 ± 9.65 | 67.49 ± 1.30 | - | - | - | - | - |
|  | C8 | 2 | 6 | 12 |  | 109.00 ± 2.35 | 122.25 ± 2.76 | 59.99 ± 0.52 | - | - | - | - | - |
